# Sex differences in the prevalence and risk factors of non-suicidal self-injury behaviors among adolescent outpatients with major depressive disorder

**DOI:** 10.3389/fpsyt.2025.1599627

**Published:** 2025-11-17

**Authors:** Guangyou Lv, Botao Ma, Siyuan Qi, Huimei An

**Affiliations:** Beijing HuiLongGuan Hospital, Peking University HuiLongGuan Clinical Medical School, Beijing, China

**Keywords:** adolescents, major depressive disorder, non-suicidal self-injury, sex differences, risk factors

## Abstract

**Objective:**

Although non-suicidal self-injury (NSSI) behaviors are particularly prevalent among adolescent outpatients with major depressive disorder (MDD), few studies have investigated the sex differences in this population. Therefore, this study aimed to investigate sex differences in the prevalence and risk factors of NSSI among adolescent outpatients with MDD.

**Methods:**

In total, 284 adolescent outpatients who met the DSM-V diagnostic criteria for MDD were recruited for this cross-sectional study. A self-designed questionnaire, the Children’s Depression Inventory (CDI), Chinese version of the Functional Assessment of Self-Mutilation (CFASM), Pittsburgh Sleep Quality Index (PSQI), and Adolescent Self-Rating Life Events Checklist (ASLEC) were employed to assess participants’ socio demographic factors, depressive symptoms, NSSI behaviors, sleep quality, and stressful life events, respectively.

**Results:**

The prevalence of NSSI behaviors was significantly higher in female adolescents than in male adolescents. Notably, female adolescents demonstrated significantly higher NSSI functioning scores, NSSI frequency, and CDI scores than male adolescents. Regression analysis showed that among female adolescents, higher PSQI and ASLEC scores were identified as significant risk factors for NSSI behaviors, with the frequency of NSSI positively correlated with ASLEC scores. However, these associations were not observed in males.

**Conclusion:**

NSSI is more prevalent in female adolescent outpatients with MDD than male outpatients with MDD. Further, there are significant sex differences in the risk factors associated with NSSI, suggesting that sex differences should be considered when developing prevention and intervention strategies for coexisting NSSI behaviors among adolescent outpatients with MDD.

## 1 Introduction

Depression is one of the most prevalent mental disorders among children and adolescents with an estimated global prevalence of 6.2% (1). The prevalence of depression among Chinese adolescents has increased, particularly in the post-COVID-19-era, with studies reporting rates as high as 20% in certain regions (2).Non-suicidal self-injury (NSSI) is defined as deliberate, self-inflicted damage to body tissues in the absence of suicidal intent. Common forms of NSSI include cutting, burning, carving, scratching, and self-hitting (3). NSSIs are associated with a range of adverse outcomes, including an increased risk of suicidal behavior, emotional dysregulation, and impaired social functioning (4). NSSI is particularly prevalent among adolescents with depression, with an estimated prevalence of 50-70% (5, 6) which not only complicates the treatment of patients with depression but also contributes to a poorer prognosis, highlighting the significance of investigating the interplay between NSSI behaviors and clinical symptoms in adolescent patients with major depressive disorder (MDD).

Recently, NSSIs among adolescents with MDD have garnered increasing attention owing to their high prevalence and severe adverse outcomes. In a study that involved 2343 adolescent inpatients and outpatients with depression from 12 hospitals across China, the detection rate of NSSI during the past year was 76.06% (7). Emerging neuroimaging evidence demonstrates widespread neurobiological alterations in adolescents with MDD and NSSI. Structural neuroimaging studies have identified microstructural abnormalities in the cingulum bundle (8) and morphological changes in the cortex (9). Functional connectivity analyses have revealed significant alterations in the neural networks associated with emotion regulation and cognitive control processes (10). Additionally, several studies have consistently identified multiple risk and protective factors for NSSI in adolescents with depression, including depression and anxiety symptoms, sleep disturbances, exposure to stressful life events, peer victimization, childhood emotional abuse, rumination, and substance or gaming addiction (11–13). Conversely, strong family relationships and psychological resilience have been identified as protective factors against NSSI (11–14).

Sex differences in the prevalence of NSSI have been extensively studied in both community and clinical samples, with most studies reporting that female adolescents have a higher rate of NSSI than male adolescents (15). For example, a recent study among school students in South Korea found that the 12-month prevalence of NSSI was 2.85 times higher in female students than in male students (16). Potential explanations for sex differences in NSSI include neurobiological, psychological, and sociocultural factors. Studies have shown that female adolescents present heightened sensitivity to negative emotions (17),exhibit greater challenges in impulse control and limited use of emotion regulation strategies (18), and are more likely to choose NSSI as a coping strategy for negative emotion regulation, whereas men are more inclined to engage in NSSI to elicit social support or communicate distress (19). A recent study reported that adolescent girls exhibit heightened capacity of facial emotion recognition, which are associated with NSSI behaviors. The increased vulnerability to depression and NSSI observed in adolescent girls may partially arise from their heightened capacity to recognize and sensitivity to the negative emotions of others (20). From a neurobiological perspective, girls typically experience earlier pubertal development than boys and demonstrate increased endocrine and physiological reactivity to stress (21). One study reported different pathological mechanisms of NSSI in male and female adolescents with depression. NSSI is more likely associated with oxidative stress responses in female adolescent patients and thyroid function in male patients with depression (22). However, sex differences in the clinical correlates associated with NSSI, particularly in adolescent outpatients with MDD, have not been thoroughly studied. Therefore, the present study aimed to investigate sex differences in the prevalence and risk factors of NSSI among adolescent outpatients with MDD. The findings contribute to a better understanding of the mechanisms underlying NSSI and to the formulation of effective prevention and intervention strategies for the population engaging in NSSI behaviors.

## 2 Method

### 2.1 Participants

This cross-sectional study continuously recruited 284 adolescent outpatients with MDD from Beijing HuiLongGuan Hospital between July 1, 2023, and July 31, 2024. Participants were included based on the following criteria: (1) Han Chinese, (2) aged 13–18 years, (3) meeting the diagnostic criteria for MDD as outlined in the fifth edition of the Diagnostic and Statistical Manual of Mental Disorders, (4) a minimum of six years of formal education, and (5) provision of written informed consent. The exclusion criteria were as follows: (1) a diagnosis of other severe mental disorders, such as schizophrenia or bipolar disorder, and (2) the presence of severe physical illnesses or substance dependence. The parents of all participants provided voluntary written informed consent, and the study protocol was approved by the Institutional Review Board of Beijing HuiLongGuan Hospital.

Data were collected using a self-administered questionnaire comprising demographic information and self-report measures. Under the guidance of trained researchers, all participants completed the questionnaire within 30-45 minutes in a hospital office. To ensure data integrity and accuracy, the researchers reviewed the complete electronic medical records and conducted supplementary interviews with family members to clarify any ambiguous or missing information.

### 2.2 Measures

The Children’s Depression Inventory (CDI) was used to assess the severity of depressive symptoms. It consists of 27 items, each of which has three statements reflecting varying levels of symptom severity. Respondents selected the statement that best described their feelings and experiences over the past two weeks. The CDI measures the key dimensions of depression, including negative mood, interpersonal problems, ineffectiveness, anhedonia, and negative self-esteem. The Cronbach’s α for the CDI was 0.86.

The Chinese version of the Functional Assessment of Self-Mutilation (CFASM) scale was used to assess the methods, frequency, and function of NSSI among adolescents (23). The scale consists of 10 different forms of the NSSI and respondents were asked to report the frequency of deliberately harming themselves on the above forms during the past 12 months. The NSSI frequency was calculated by summing the frequencies of the ten NSSI forms. The overall frequency of NSSI was divided into five levels: 0 times (0 = never), 1–12 times (1 = rarely), 13–36 times (2 = occasionally), 37–96 times (3 = often), and >96 times (4 = frequently). The NSSI functions consist of 15 items rated on a four-point Likert scale. The Cronbach’s α of the scale was 0.89.

The Pittsburgh Sleep Quality Index (PSQI) was employed to evaluate sleep quality. It consists of 19 items that are combined to produce seven component scores, each ranging from 0 to 3. The component scores were subsequently summed to produce a global PSQI score. Higher scores reflect poorer sleep quality, with a global score more than 5 indicating significant sleep disturbances. The Cronbach’s α for the PSQI was 0.81.

The Adolescent Self-rating Life Events Checklist (ASLEC) was used to measure the impact of stressful life events on adolescents. It is a self-reported questionnaire that asks adolescents to identify and rate the severity of various life events that they have experienced over the past six to twelve months. The Cronbach’s α for the ASLEC was 0.86.

### 2.3 Statistical analysis

Statistical analyses were performed using IBM SPSS Statistics 22.0. Data are presented as mean (standard deviation, SD) for continuous variables and as frequencies (proportions) for categorical variables. Sex differences in demographic and clinical characteristics were tested using the t-test for continuous variables or the chi-square test for categorical variables. Binary logistic regression was used to explore the risk factors of NSSI in male and female adolescents. Spearman’s correlation analysis was used to evaluate the correlation between NSSI frequency and the participants’ demographic and clinical characteristics. Finally, ordinal logistic regression was used to identify the important variables associated with NSSI frequency in male and female adolescents. All statistical analyses were conducted using two-tailed tests, with a significance threshold set at P < 0.05.

## 3 Results

### 3.1 Sex differences in the prevalence of NSSI behaviors among adolescent outpatients with MDD

This study recruited 284 adolescent outpatients with depression, including 201 with NSSI behavior (44 male adolescents and 158 female adolescents) and 82 without NSSI behavior (36 male adolescents and 46 female adolescents). The prevalence of NSSI among the adolescent outpatients with depression was 71.1%. Female adolescents had a significantly higher prevalence rate of NSSI behaviors than male (77.4% Vs 55.0%, χ2 = 7.052, p=0.008).

### 3.2 Differences in demographic and clinical characteristics among adolescent MDD outpatients with and without NSSI behaviors

The differences in demographic and clinical characteristics between the groups with and without NSSI behaviors are shown in **Table 1**. In the NSSI behavior group, the proportion of female adolescents (78.2% Vs 56.0%), CDI scores (32.4 ± 8.4 Vs 24.7 ± 8.3), PSQI scores (10.5 ± 4.5 Vs 6.1 ± 3.3) and ASLEC scores (62.2 ± 16.4 Vs 46.0 ± 8.3) were significantly higher than in the without NSSI behavior group, all p<0.05. No significant differences were observed between the two groups in any other demographic or clinical characteristic variables (all p > 0.05).

**Table 1:** The differences in demographic and clinical characteristics between with and without NSSI behavior groups.

Variables	With NSSI group (n=202)	Without NSSI group (n=82)	F/χ^2^	P
Age (years)	15.0 ± 1.6	15.0 ± 1.7	-0.032	0.751
Sex (M/F)	44/158	36/46	7.052	0.013
Education (years)	9.1 ± 2.2	9.1 ± 1.9	-0.034	0.973
BMI(kg/m^2)^	22.2 ± 4.6	22.9 ± 4.9	-0.886	0.624
Han Chinese (Y/N)	88/13	35/4	5.174	0.075
Smoke (Y/N)	11/89	2/39	1.302	0.346
Schooling (Y/N)	54/32	20/14	0.162	0.683
Only child (Y/N)	39/49	14/20	0.099	0.84
Age of onset (years)	13.2 ± 2.0	13.0 ± 2.5	0.494	0.622
Duration of disease (years)	2.0 ± 1.2	2.4 ± 2.8	-1.207	0.23
Number of hospitalizations	1.1 ± 0.12	1.2 ± 0.9	-0.462	0.645
CDI scores	32.4 ± 8.4	24.7 ± 8.3	4.890	<0.001
PSQI scores	10.5 ± 4.5	6.1 ± 3.3	5.580	<0.001
ASLEC scores	62.2 ± 16.4	46.0 ± 8.3	5.718	<0.001

M/F, male/female; Y/N, Yes/No, CDI, Children’s Depression Inventory; PSQI, Pittsburgh Sleep Quality Index; ASLEC, Adolescent Self-rating Life Events Checklist.

### 3.3 Sex differences in demographic, NSSI, and clinical characteristics among adolescent MDD outpatients with NSSI behaviors

Sex differences in the demographic and clinical characteristics of adolescent depression outpatients with NSSI behaviors are shown in **Table 2**. Female adolescents had significantly higher CDI scores (33.5 ± 8.5 Vs 28.5 ± 7.1), NSSI functioning scores (38.0 ± 11.5 Vs 29.8 ± 11.6), and NSSI frequency (3.3 ± 1.4 Vs 2.6 ± 1.4) than male adolescents (all p > 0.05). No sex differences were found in the other demographic or clinical characteristics (all p > 0.05).

**Table 2:** Gender differences in demographic, clinical, and NSSI characteristics among adolescent depression outpatients with NSSI behaviors.

Variables	Male n=44	Female n=158	F/χ^2^	P
Age (years)	15.2 ± 1.7	14.8 ± 1.7	0.754	0.453
Education (years)	9.0 ± 2.0	9.1 ± 2.3	-0.139	0.889
BMI (kg/m^2)^	21.8 ± 5.2	22.3 ± 4.4	-0.43	0.668
Age of onset (years)	13.0 ± 1.8	13.2 ± 2.1	-0.406	0.686
Duration of disease (years)	2.2 ± 1.5	2.0 ± 1.2	0.754	0.473
Number of hospitalizations	1.1 ± 1.0	1.1 ± 1.2	-0.164	0.870
CDI scores	28.5 ± 7.1	33.5 ± 8.5	-2.501	0.014
PSQI scores	10.1 ± 5.3	10.6 ± 4.3	-0.534	0.595
NSSI function scores	29.8 ± 11.6	38.0 ± 11.5	-2.947	0.004
NSSI frequency	2.6 ± 1.4	3.3 ± 1.4	-2.216	0.029
ASLEC scores	57.4 ± 17.4	63.6 ± 16.0	-1.557	0.123

CDI, Children’s Depression Inventory; PSQI, Pittsburgh Sleep Quality Index; ASLEC, Adolescent Self-rating Life Events Checklist.

### 3.4 Sex differences in risk factors for NSSI behaviors among adolescent outpatients with MDD

Binary logistic regression analysis identified that PSQI (OR = 2.514, 95% CI: 1.065-5.934, p = 0.035) and ASLEC scores (OR = 1.082, 95% CI: 1.025-1.141, p = 0.004) as significant risk factors for NSSI among female adolescents (**Table 3**). In contrast, no significant associations were found for males.

**Table 3:** Binary logistic regression of risk factors for NSSI in female adolescents.

Independent variables	β	S.E	Walds	*P*	OR (95% CI)
Age	-0.203	0.465	0.191	0.662	0.816 (0.328, 2.03)
Education	0.437	0.291	2.26	0.133	1.548 (0.876, 2.736)
Age of onset	-0.084	0.289	0.085	0.771	0.919 (0.522, 1.619)
Duration of disease	-0.031	0.152	0.042	0.837	0.969 (0.72, 1.305)
CDI scores	-0.037	0.05	0.533	0.465	0.964 (0.874, 1.064)
ASLEC scores	0.078	0.027	8.187	0.004	1.082 (1.025, 1.141)
PSQI scores	0.922	0.438	4.424	0.035	2.514 (1.065, 5.934)

CDI, Children’s Depression Inventory; PSQI, Pittsburgh Sleep Quality Index; ASLEC, Adolescent Self-rating Life Events Checklist; CI, Confidence Intervals

### 3.5 Sex differences in the correlation of the demographic and clinical correlates with NSSI frequency

Spearman’s correlation analysis revealed that among male adolescents, the NSSI frequency exhibited a significant positive correlation with the duration of illness (r=0.613, p=0.003). In contrast, NSSI frequency was significantly positively correlated with CDI (r=0.248, p=0.028) and ASLEC scores (r=0.399, p<0.001) in female adolescents. Furthermore, an ordinal logistic regression analysis with NSSI frequency as the dependent variable and age, education, age of onset, duration of illness, CDI scores, PSQI scores, and ASLEC scores as the independent variables revealed that ASLEC scores (OR = 1.046, 95% CI: 1.012-1.082, p=0.007) were significantly associated with NSSI frequency in female adolescents (**Table 4**). In contrast, no variables remained significant in the final model for males.

**Table 4:** Ordinal logistic regression of risk factors for NSSI frequency in female adolescents.

Independent variables	β	S.E	Walds	*P*	OR (95% CI)
Age	0.325	0.379	0.734	0.392	1.384 (0.658, 2.907)
Education	-0.158	0.163	0.934	0.334	0.854 (0.620, 1.176)
Age of onset	-0.253	0.274	0.852	0.356	0.776 (0.454, 1.328)
Duration of disease	0.01	0.311	0.001	0.973	1.010 (0.549, 1.859)
CDI scores	0.027	0.028	0.927	0.336	1.027 (0.972, 1.084)
ASLEC scores	0.045	0.017	7.189	0.007	1.046 (1.012, 1.082)
PSQI scores	0.058	0.062	0.872	0.35	1.060 (0.938, 1.197)

CDI, Children’s Depression Inventory; PSQI, Pittsburgh Sleep Quality Index; ASLEC, Adolescent Self-rating Life Events Checklist; CI, Confidence Intervals.

## 4 Discussion

This study revealed that over 70% of Chinese adolescent outpatients with MDD exhibited NSSI behaviors. Notably, female adolescents demonstrated a significantly higher prevalence of NSSI, NSSI functioning scores, NSSI frequency, and CDI scores than male adolescents. Furthermore, significant sex differences were observed in the risk factors associated with NSSI. Among female adolescents, higher PSQI and ASLEC scores were identified as significant risk factors for NSSI behaviors, with the frequency of NSSI positively correlated with ASLEC scores. However, these associations were not observed in males.

The present study found that more than 70% of adolescent outpatients with MDD exhibited NSSI behaviors, which was significantly higher than that reported in nonclinical samples, where approximately 22% of adolescents engage in NSSI behaviors (24). This finding is consistent with previous research on adolescents with psychiatric disorders. For example, a cross-sectional study conducted among psychiatric inpatients aged 10–19 years in China revealed that 77% of these adolescents exhibited NSSI behaviors (25). Similarly, another study from China found that 62% of inpatients with depression or bipolar disorder had a history of NSSI (26) and a survey from Germany indicated that approximately 60% of psychiatric inpatients engage in NSSI behaviors (27). Furthermore, a recent meta-analysis revealed that among adolescents with depression, the lifetime prevalence of NSSI was 52%, whereas the period prevalence was 57% (6). Collectively, these studies suggest that the prevalence of NSSI behaviors is significantly higher among adolescents with psychiatric disorders, particularly those with depression, compared to the general adolescent population.

Consistent with previous studies, the present study also observed that female adolescents exhibited a higher prevalence of NSSI behaviors, NSSI frequency, and NSSI functioning scores than male adolescents with MDD. For example, a previous meta-analysis reported that the lifetime prevalence of NSSI was higher in female adolescents than in male adolescents among nonclinical samples (28). Similarly, a recent study reported that sex was significantly associated with NSSI, with 78% of female adolescents engaging in NSSI behaviors compared to 57% of male adolescents among adolescents diagnosed with depressive or bipolar disorders (29). Another study reported that female adolescent patients with MDD had a higher frequency of NSSIs than male patients (30).

The present study observed significant sex differences in the risk factors associated with NSSI among adolescent outpatients with MDD. Specifically, among female adolescents, higher PSQI and ASLEC scores were identified as significant risk factors, and NSSI frequency was positively correlated with ASLEC scores. However, these associations were not observed in males. A previous study suggested that insomnia and NSSI are bidirectionally linked via depressive symptoms in adolescents (31). Chronic insomnia or sleep deficiency in adolescents may increase emotional distress, impair their ability to regulate negative emotions, and reduce inhibition of negative information (32, 33). These factors make it difficult to initiate and maintain efforts in positive emotion regulation, exacerbating their distress and leading them to engage in NSSI to avoid adverse emotional experiences (34). Consistent with the findings of the present study, a Swedish study investigated the relationship between poor sleep and NSSI in 881 adolescents and revealed that poor sleep was prospectively associated with NSSI in girls, but not in boys. Specifically, 77% of girls who reported poor sleep at baseline exhibited repeated NSSI one year later (35). A possible explanation may be that female adolescents are disproportionately affected by interactions between hormonal cycles and psychosocial stressors (21), which amplify their vulnerability to insomnia. Furthermore, sleep problems often co-occur with rumination, a maladaptive cognitive style that is more prevalent in female adolescents, wherein prolonged negative thinking exacerbates emotional distress and motivates NSSI as a maladaptive coping mechanism (36). These studies suggest that screening for poor sleep in adolescents could help identify girls at risk for NSSI, and that improving poor sleep may help reduce NSSI behaviors in female adolescents with MDD.

In the present study, ASLEC scores were another risk factor for NSSI in female adolescents, and NSSI frequency was positively correlated with ASLEC scores. Several studies have highlighted the role of stressful life events, such as academic pressure, family conflicts, and peer victimization, in the development and maintenance of NSSI, particularly among adolescents (37–40). Consistent with the findings of the present study, a recent cross-sectional study investigated the impact of life events and emotional stress on NSSI among psychiatric adolescent inpatients aged 10-19 years, and found a higher risk of NSSI among younger female adolescents, those with a suicide history or depression symptoms, and those with higher ASLEC scores (25). Another prospective longitudinal community-representative study investigated the contribution of stressful life events to NSSI in adolescents from the community and found that female adolescents are more likely than male adolescents to use NSSI behaviors when faced with a stressful event in the context of school and intimate relationships; the risk of NSSI in female adolescents increases with each additional life event during ages 13-17. However, this pattern does not hold for male adolescents (38). A possible explanation may be that female adolescents may experience a more pronounced cumulative impact of stressful life events on psychosis, coupled with heightened emotional reactivity to daily life stressors compared to male adolescents (41), as well as a more rapid HPA axis response with a greater output of steroid hormones (42). According to Nock’s theory (3), NSSI is a maladaptive coping mechanism in response to stress. Stressful life events and their accumulation may evoke over-arousal in female adolescents, imposing demands that exceed their coping capacities. Stressful life events exacerbate these overwhelming demands. Consequently, the inability to manage these stressors may lead to feelings of overwhelm, which in turn can trigger NSSI as a maladaptive strategy for alleviating psychological distress. In contrast, male adolescents exhibit more maladaptive coping behaviors, such as substance use or aggressive behaviors, in response to moderate levels of stress (15). All these studies suggest that early identification of adolescents, particularly girls, with high ASLEC scores could facilitate targeted interventions aimed at reducing stress and improving emotional regulation skills, ultimately reducing the risk of NSSI.

This study has some limitations. First, the reliance on self-report measures may introduce recall and social desirability biases, potentially compromising the accuracy of the findings. Second, while the analysis identified key risk factors for NSSI, it did not account for potential confounding variables such as family socioeconomic status (e.g., parental income and education) and peer relationship dynamics, which may independently or interactively influence NSSI behaviors. Third, the cross-sectional design does not explain the causal relationship between risk factors and NSSI behaviors. Finally, the identification of significant factors associated with NSSI was precluded in male adolescents with MDD, likely due to the limited sample size. Future investigations should employ longitudinal designs with expanded samples to delineate causal relationships and the dynamic interactions among biological, psychological, and environmental factors over time, while incorporating objective data collection methods (e.g., behavioral assessments and physiological markers) to minimize reporting biases. Additionally, expanding the scope of the variables to include familial, socioeconomic, and cultural determinants could refine our understanding of sex-specific pathways to NSSI in outpatients with adolescent depression.

This study demonstrated that NSSI is more prevalent in female adolescents than in male adolescents among adolescent outpatients with MDD and that there are significant sex differences in the risk factors associated with NSSI, suggesting that these differences should be considered when developing and implementing prevention and intervention strategies for coexisting NSSI behaviors among adolescent outpatients with MDD.

## Data Availability

The raw data supporting the conclusions of this article will be made available by the authors, without undue reservation.
